# Estimating the mental health costs of racial discrimination

**DOI:** 10.1186/s12889-016-3868-1

**Published:** 2016-11-29

**Authors:** Amanuel Elias, Yin Paradies

**Affiliations:** Alfred Deakin Institute for Citizenship & Globalization, Faculty of Arts and Education, Deakin University, 221 Burwood HWY, Burwood, VIC 3125 Australia

**Keywords:** Burden of disease, DALY, Health cost, Racial discrimination, Australia

## Abstract

**Background:**

Racial discrimination is a pervasive social problem in several advanced countries such as the U.S., U.K., and Australia. Public health research also indicates a range of associations between exposure to racial discrimination and negative health, particularly, mental health including depression, anxiety, and post-traumatic stress disorder (PTSD). However, the direct negative health impact of racial discrimination has not been costed so far although economists have previously estimated indirect non-health related productivity costs. In this study, we estimate the burden of disease due to exposure to racial discrimination and measure the cost of this exposure.

**Methods:**

Using prevalence surveys and data on the association of racial discrimination with health outcomes from a global meta-analysis, we apply a cost of illness method to measure the impact of racial discrimination. This estimate indicates the direct health cost attributable to racial discrimination and we convert the estimates to monetary values based on conventional parameters.

**Results:**

Racial discrimination costs the Australian economy 235,452 in disability adjusted life years lost, equivalent to $37.9 billion per annum, roughly 3.02% of annual gross domestic product (GDP) over 2001–11, indicating a sizeable loss for the economy.

**Conclusion:**

Substantial cost is incurred due to increased prevalence of racial discrimination as a result of its association with negative health outcomes (e.g. depression, anxiety and PTSD). This implies that potentially significant cost savings can be made through measures that target racial discrimination. Our research contributes to the debate on the social impact of racial discrimination, with implications for policies and efforts addressing it.

## Background

### Introduction

Existing scholarship acknowledges the cost of racial discrimination and its disproportionate bearing on racial minorities [1–3]. However, the economic consequences experiences of racial discrimination (EOD) extend beyond the immediate targets to those witnessing discrimination [4] and even to the perpetrators themselves [5–7]. Flow on effects can occur for the targets’ immediate and/or extended families [8–10], communities, and government institutions that are tasked with combating discrimination. At an aggregate level, the country can incur losses in terms of direct healthcare expenditures arising from the health-related impact of racism and indirectly in the form of forgone output related to employment discrimination.

The purpose of this study is to cost the negative mental health outcomes associated with exposure to EOD. We apply a step by step costing method to estimate the health impact of EOD, focusing on four key health outcomes including depression, anxiety, post-traumatic stress disorder (PTSD) and psychological disorder.

In this and the next section, we discuss related literature and the methods involved including the data we utilise in our analysis. Then, we present the results reporting burden of disease estimates, DALYs and health cost estimates. We also report a sensitivity analysis, taking three scenarios, each assuming alternative parameters. We then discuss the findings, before presenting a brief conclusion.

### Related research

Although there has been research on employment-related costs of discrimination [11], intangible costs that manifest in terms of pain and suffering arising from the physical and psychological illnesses may also be considerable. A wide body of evidence has documented the negative health outcomes associated with experiences of racial discrimination (EOD) [12–16]. However, to our knowledge, no previous study has so far estimated these health-related costs in monetary terms. In this study, we attempt to fill this gap by estimating the dollar value of these intangible health related costs from a societal perspective, focusing, particularly, on mental illnesses. Using Australian prevalence data and association data from a recent meta-analysis [14], we calculate burden of disease (BoD) estimates in terms of disability adjusted life years (DALYs) for outcomes with the requisite data.

Previously, estimations of the economic cost of EOD followed the neoclassical approach which generally focuses on indirect costs related to productivity loss as a result of discrimination premiums [17, 18]. In labour economics, these costs are measured as differentials in wages, earnings, employment, promotion and labour supply (see [19–21] for reviews). Similarly, literature at the intersection of law and economics has been limited to detecting evidence of racial discrimination rather than estimating its human and social impact. Another literature related to the housing market, measures EOD-related costs to minority group customers in terms of potential indirect costs, associated with higher prices and limited services [22, 23].

So far, only a handful of studies have gone beyond a focus on the indirect microeconomic impact in estimating the cost of EOD. Although these studies are also based on indirect productivity loss (i.e., opportunity costs to businesses), they have attempted to estimate the potential aggregate effect of EOD in an economy [24–26]. The estimations attribute the costs to foregone wages for minorities, and indirectly measure losses in national output from an industry perspective.

### Health cost estimation

Depending on the prevalence of EOD and the degree of association between EOD and health outcomes, EOD can also be seen as a public health issue as it can affect the overall wellbeing and quality of life of a society. This aspect of the cost of EOD can be assessed using cost of illness methods which are widely utilised in health economics. Using this approach, the cost of EOD can be conceptualised in terms of health outcomes from an individual/societal perspective where the health burden is largely borne by individuals, and cumulatively by a society, in the form of physical and mental illnesses.

Following the cost of illness literature [27–29], the cost of EOD can either be determined by identifying the cost components from different perspectives (industry/business, government, or society) or classified into three distinct components: direct costs, indirect costs and intangible costs. Taking the latter classification, the direct cost component of EOD would include all the health related costs that are immediate consequences of discrimination. These costs include health sector costs such as outpatient costs, prescription drug purchases, medical fees etc. Other direct cost components include those borne by different sectors of an economy including production and consumption related costs, administrative, welfare/transfer and other costs.

Indirect costs involve productivity loss and other employment related costs associated with EOD. The loss in productivity includes, for instance, the costs incurred by employers due to absenteeism, resulting from the target having to cope with depression, physical illness, hospital visits etc. In addition, costs due to lower performance at work by employees exposed to discrimination are included. Potentially, however, the largest amount of illness cost due to EOD would come from the intangible cost component. In this paper, we estimate this aspect of the health cost of EOD. Intangible costs involve the pain, suffering and premature death associated with a risk factor/health outcome. For example, depression and related symptoms associated with EOD affect the wellbeing of targets. This and other associated illnesses can be included in the estimation of loss in wellbeing in terms of disability adjusted life years (DALYs), the tool widely used in costing a range of epidemiological conditions [27, 30]. This tool allows us to determine the burden of disease (BoD) fractions of illnesses arising from the targets’ exposures to discriminatory episodes.

## Methods

### Data

In this study, the individual target is the unit of reference. We use Australian self-report data from three nationally representative surveys (the Scanlon Foundation’s Mapping Social Cohesion (MSC) survey, the Challenging Racism Project (CRP) and the 2012 National Dual-frame Omnibus Survey (DFO)) to estimate the prevalence of EOD. We utilise these prevalence data along with the magnitude of association between racism and four key health outcomes from a recent comprehensive meta-analysis [14] to calculate BoD estimates ascribed to EOD through loss of DALYs. We then use the results to compare the current health status with a counterfactual of no lifetime discrimination. Finally, we convert these estimates into monetary estimates using standard parameters. We then conduct sensitivity analysis for the robustness of the cost estimations, using three scenarios of the valuation of life (low, medium and high value of statistical life - VSL) and a range of discount rates.

#### Prevalence of EOD and its association with health

Racial discrimination is usually measured either based on self-report or observational data [31]. One advantage of self-report data in discrimination research is that it enables researchers to directly measure the experiences of the targets [32–34]. Unlike observational data, it does not rely on observed differentials in the outcomes (e.g. wages and salaries, years of schooling etc.) of different racial groups per se to determine the prevalence of EOD. Self-report data can extract more complete data on EOD as perceived by targets themselves. However, self-report data can be susceptible to reporting bias [35]. Respondents may not correctly understand the question; they may over report their experiences (vigilance bias); or they may underreport them (minimisation bias). Yet, research to-date indicates that under- rather than over-reporting is more prevalent, in relation to racial discriminaton [36, 37]. Noting the strengths and drawbacks of self-report data, we utilise the three national surveys mentioned above to estimate EOD among Australian respondents.

### Measures

#### Prevalence of EOD in Australia

Our first EOD measure is from the CRP survey (*n* = 12,512) which consists a series of surveys conducted between 2001 and 2008 in different states in Australia [38]. The EOD measure from this study has six items with 5-point Likert type responses where 22.3% of the respondents reported experiencing racial discrimination. The second dataset, the MSC survey (*n* = 2000 in each wave), was conducted between 2007 and 2013 [39]. A dichotomous EOD measure in this survey indicates that 9–19% of respondents reported EOD over the period 2007–13. The DFO survey (2012, *n* = 2000), another Australian dataset conducted by the Social Research Centre, contains three items on racism as well as general health, physical activity, smoking, and substance use. Responses were coded 1 = yes and 2 = no, and almost 20% of the respondents indicated experiencing racial discrimination.

The prevalence data we utilise in this study classifies respondents first by gender, then by age into seven age categories, ranging 18–100 years. Table 1 below summarises the distribution of EOD in the three datasets based on age and gender. To generate a weighted average EOD data from the three national surveys, we first dichotomised the 5-point CRP EOD data, before decomposing the result by age and gender. EOD responses including “sometimes” and over were recoded as “1” while the rest were recoded as “0”. The last two columns of Table 1 indicate that the average prevalence of EOD among those aged 64 years and below ranged 13.4 to 20.1%.

**Table 1:** Prevalence of racial discrimination in Australia by age and gender

Age group	Gender							
	CRP		MSC		DFO		Weighted average	
	Males	Females	Males	Females	Males	Females	Males	Females
17–24	32.7	24.8	20.0	14.6	18.8	23.5	23.8	20.1
25–34	33.6	21.8	16.5	13.4	25.7	32.5	22.3	19.0
35–44	28.5	21.8	13.2	12.9	22.7	23.7	18.5	18.0
45–54	26.6	19.9	12.7	11.6	13.8	23.4	17.0	16.5
55–64	24.8	15.6	7.9	9.7	17.4	20.1	13.7	13.4
65–74	14.8	10.7	5.2	5.7	10.0	16.8	8.4	9.0
75–100	9.7	7.2	5.1	0.6	4.7	13.3	6.4	4.8

Note: Values are percentages of those who indicated they experienced racial discrimination in their lifetime. The last two columns are estimated based on the weighted average from CRP, MSC & DFO data

#### Association between EOD and health outcomes

A comprehensive assessment of the overall cost of EOD requires estimates of the health impact of exposure to EOD. As part of a recent meta-analysis, we investigated 293 studies, and found significant unadjusted associations between EOD and some key health outcomes, ranging from depression to hypertension, based on studies published until October 2013 [14]. Subject to our inclusion and exclusion criteria, we identified more than 21 associations of EOD and major health outcomes.^1^ However, in this study, we focus on only four illnesses across 102 studies, because the rest are either co-morbid with illnesses such as depression, or they are not available in the Global Burden of Disease which is the basis of our analysis. For example, chronic stress is associated with depression, and depression in turn is associated with obesity [40, 41].

In most cases, the studies we included utilise standard measures. For example, the instruments used in depression studies include the Centre for Epidemiological Studies Depression scale, the Major Depression Episodes, and the Beck Depression Inventory. For anxiety, studies utilised instruments such as the State Trait Anxiety Index, the Beck Anxiety Inventory and the General Health Questionnaire.

To allow an unbiased estimation of BoD attributable to EOD, the ideal effect size would be risk ratio (RR), indicating the prevalence rate of the health outcome among the exposed group relative to the unexposed group. However, almost all of the included studies (94.1%) had insufficient data for the calculation of RR. Given sufficient data, it is possible to convert odds ratios (OR) to RR using the formula proposed by Zhang & Yu [42]. However, the prevalence of the outcome on the control (unexposed) group was not reported for most of the studies reporting OR (and other OR convertible estimate). Therefore, only associations based on ORs (*n* = 197) were utilised in this paper, resulting in a focus on 101 studies (reported in 128 articles) involving 83,057 participants in total. There is a limitation in using OR in BoD analysis in that it exaggerates the risk of the outcome if the outcome of interest is common [43, 44]. However, Davies et al. [43] indicate that the size of the exaggeration depends on the size of the OR. Smaller OR would have minor exaggeration effect compared to large OR, therefore, using OR as alternative for RR would introduce minor exaggeration for OR < 3.0. In this study, the OR for all the illnesses except PTSD is less than three. Although we expect the OR for PTSD to have some exaggeration effect, the impact on the overall study findings is negligible given the small prevalence rate of PTSD considered here along with the small disability weight assigned to it in the GBD.

Within this association data, one issue that remained is the heterogeneity in the measures where the outcome scales varied from dichotomous (yes/no) categories to a range of categories including 3 to 9-point Likert type scales, and in some cases continuous measures. To account for this heterogeneity, we report two types of response categories. First, we report health outcomes for dichotomous response measure as one group with associations comparing the EOD-exposed and unexposed respondents. Second, we weight average the rest of the exposure measures and report them as one group comparing those who had low with those who had high level of EOD exposure (see Table 2). The sample size reported in Table 2 varies by the number of studies included, with depression having the largest overall sample (*n* = 124,049) and PTSD having the least overall sample (*n* = 2621). All associations were statistically significant (highly so in most cases) with odds ratios of two to three for all health outcomes except for PTSD.

**Table 2:** List of association between EOD and health outcomes categorised by major illnesses

Illness/Health outcome	Statistical outcome						
	Odds ratio	Lower limit (95% CI)	Upper limit (95% CI)	*Z*-value	*P*-value	Total sample size	Number of associations
A. Exposure response: yes/no							
Anxiety	2.050	1.758	2.391	9.140	0.000	13,216	13
Depression	2.051	1.811	2.323	11.319	0.000	70,115	32
PTSD	4.560	2.656	7.829	5.501	0.000	212	2
Psychological disorders	2.584	2.068	3.137	9.318	0.000	13,705	3
B. Exposure response: 3-point and over							
Anxiety	2.275	1.619	3.279	4.922	0.019	11,228	37
Depression	2.386	1.987	2.870	9.499	0.005	53,115	90
PTSD	3.756	2.225	6.637	5.248	0.040	2021	9
Psychological disorders	2.018	1.445	2.944	4.793	0.030	3946	11

Note: This table reports the effect size of the association between EOD and health outcomes categorised by response types from a meta-analysis [14]. Panel A is based on a dichotomous measure of EOD while panel B is based on EOD measured as a categorical variable with 3-point and over response options

### Statistical analysis

#### DALYs, PAF and health costs

BoD analyses of risk factors/health outcomes are frequently used in measuring the health impact of a range of exposures. The health impact of EOD can likewise be estimated using this method. The World Health Organisation (WHO) provides guideline for the calculation of BoD. Following this guideline, BoD can be defined as a measure quantifying ‘the gap between a population’s current health and an ideal situation where everyone lives to old age in full health’ [29, p. 4]. It is numerically estimated in terms of DALYs where a loss of one DALY is equivalent to a loss of one healthy year of life [45]. Mathers et al. [46, p. 3] describe DALY as ‘a summary measure of population health that combines in a single indicator, years of life lost from premature death and years of life lived with disabilities.’ Several international studies have used BoD based on DALY in cost estimations for a range of illnesses [47–49].

Although BoD represents direct health impact resulting from the exposure, it is not a measure of immediate expenditures. It exists as an opportunity cost that was lost or a cost that can be averted. In different cost of illness studies, DALYs express the ‘pain, suffering and premature mortality’ component of the health costs. In this paper, prevalence data is used to estimate these costs attributable to racial discrimination. Either prevalence or incidence measures are used in the literature in the calculation of the BoD [45, 47]. Formally, DALY is calculated as:

1 $$ DALY\kern0.5em =\kern0.5em \mathrm{Years}\ \mathrm{of}\ \mathrm{Life}\ \mathrm{Lost}\kern0.75em +\kern0.75em  Years\  Lived\  with\  Disability $$

where years of life lost is premature mortality due to exposure to the risk factor/illness and years lived with disability is the number of years of less than functional life attributable to the risk factor/illness. In theory, exposure to EOD is causally associated with some illnesses which are in turn causes of mortality (e.g., anxiety and depression, see: [47]). Due to the timing issue regarding the exposure to EOD and the incidence/prevalence of death, the causal relationship is likely to be confounded. In addition, mortality is already excluded from the meta-analysis which is the basis of our analysis, as racial discrimination per se appears to have no immediate association with mortality [50]. Therefore, assuming years of life lost = 0 in this analysis, the measure to be estimated becomes:

2 $$ DALY\kern0.5em =\kern0.5em  Years\  Lived\  with\  Disability $$

where:

3 $$ YLD\kern0.5em =\kern0.5em  Prevalence\kern0.5em *\kern0.5em  Duration\kern0.5em *\kern0.5em  Weight $$

where ‘prevalence’ is the prevalence of the illness, ‘duration’ refers to the duration of the illness since its onset, and ‘weight’ is the disability weight. To exploit the population attributable fraction (PAF), data already estimated in this paper, an alternative specification is used to estimate the DALYs [51]. The duration of illness is replaced by the PAF whereby the value will be interpreted as DALYs attributable to the EOD as a risk factor. This is specified by:

4 $$ DALY\kern0.5em =\kern0.5em  Prevalence\kern0.5em \ast \kern0.5em  Weight\kern0.5em \ast \kern0.5em PAF $$

where PAF indicates the fraction measuring the degree of causal relationship between EOD and the illness. The PAF or BoD attributable to a risk factor (e.g., EOD) is estimated using the standard formula:

5 $$ PAF=\left[{\displaystyle \sum_{i=0}^k}{p}_i\left(R{R}_i-1\right)\right]\div \kern0.5em \left[{\displaystyle \sum_{i=0}^k}{p}_i\left(R{R}_i-1\right)+1\right] $$

where $i$ stands for the exposure category and $i=0$ represents the baseline category (no exposure); ${p}_i$ is the prevalence of the exposure (risk factor) for the *i*th category; $R{R}_i$ is the relative risk for the group with $i$ level of exposure and is compared with no exposure. After some algebraic manipulation and assuming the relative risk for the unexposed group to be 1, this formula can be re-written as:

6 $$ PAF=\left[{\displaystyle \sum_c}{p}_cR{R}_c-1\right]\kern0.5em \div \kern0.5em \left[{\displaystyle \sum_c}{p}_cR{R}_c\right] $$

where $c$ stands for the category of interest. Since most studies included for association estimation have no sufficient data to calculate relative risk (RR) ratio for an outcome, the formula we actually utilise in calculating the PAF is:

7 $$ PAF=\left[{\displaystyle \sum_c}{p}_cO{R}_c-1\right]\kern0.5em \div \kern0.5em \left[{\displaystyle \sum_c}{p}_cO{R}_c\right] $$

Once we estimated the DALYs attributable to the prevalence of EOD, the next step is to convert them to monetary value which we do so using the value of statistical life (VSL) approach. A range of values have been suggested in the literature as conversion parameters [52–55]. These studies suggest a VSL value ranging between 0.6 million and $19.1 million. A recent study by Andersson and Treich [56] reports a wider range of a VSL of $261 thousand to $36 million. The corresponding cost per DALY falls in the range of $70,000-$175,000. Drawing from the literature, Access Economics [57] uses a VSL of $3.7–9.6 million in measuring the cost of domestic violence in Australia. This corresponds to a conversion rate of $162,561 per DALY. In this analysis, we use a rate close to this ($166,250) as it draws from available evidence and best practice in an Australian context. The conversion involved key parameters including a VSL of $6.65 million, a 3.3% discount rate, and a timeframe of 40 years. Our conversion rate is estimated using the discounting formula:

8 $$ VSL={\displaystyle \sum}\frac{Value\  of\  Life\  Years}{{\left(1+r\right)}^t} $$

where $r$ is the discount rate. Value of life years is multiplied by DALY for each health outcome to convert the latter to a monetary value that represents the health cost attributable to the risk factor, an estimation we report in the next section.

## Results

### Population attributable fractions

The third stage of our analysis combines the prevalence measure of EOD and the corresponding association effect sizes we use to estimate population attributable fractions (PAFs). Tables 3 and 4 report the EOD related PAFs for males and females, for each of the illnesses which have statistically significant association with EOD. These tables report the proportion of the prevalence of the designated illness attributable to the prevalence of EOD. Table 3, which was calculated assuming no exposure to EOD, indicates that reducing EOD to zero can result in a 20% reduction in the prevalence of depression among men aged 17–24. A similar reduction of EOD can result in an 18.7% reduction in the prevalence of stress among women aged 35–44. The rest of the values can be interpreted likewise.

**Table 3:** Population attributable fractions: the prevalence of illness attributable to EOD (dichotomous) by age and gender

Age group	Illness/Health outcome							
	Depression		Anxiety		PTSD		Psychological disorders	
	PAF (%)	95% CI	PAF (%)	95% CI	PAF (%)	95% CI	PAF (%)	95% CI
Males								
17–24	20	(16.2–23.9)	20	(15.3–24.9)	45.9	(28.3–61.9)	27.4	(20.3–33.7)
25–34	19	(15.3–22.8)	19	(14.4–23.7)	44.2	(26.9–60.3)	26.1	(19.2–32.2)
35–44	16.2	(13.0–19.6)	16.2	(12.3–20.4)	39.6	(23.4–55.8)	22.6	(16.5–28.3)
45–54	15.2	(12.1–18.4)	15.1	(11.4–19.1)	37.7	(22.0–53.7)	21.2	(15.4–26.6)
55–64	12.6	(10.0–15.3)	12.6	(9.4–16.0)	32.7	(18.5–48.3)	17.8	(12.7–22.6)
65–74	8.1	(6.4–10.0)	8.1	(6.0–10.5)	23	(12.2–36.5)	11.8	(8.2–15.2)
75–100	6.3	(5.0–7.9)	6.3	(4.7–8.2)	18.7	(9.6–30.6)	9.3	(6.4–12.1)
Females								
17–24	17.4	(14.0–21.0)	17.4	(13.2–21.8)	41.7	(25.0–57.8)	24.1	(17.7–30.0)
25–34	16.6	(13.3–20.1)	16.6	(12.6–20.9)	40.3	(23.9–56.4)	23.1	(16.8–28.8)
35–44	15.9	(12.7–19.2)	15.9	(12.0–20.0)	39	(22.9–55.1)	22.2	(16.1–27.7)
45–54	14.8	(11.8–17.9)	14.8	(11.1–18.6)	37	(21.4–53.0)	20.7	(15.0–26.0)
55–64	12.3	(9.8–15)	12.3	(9.2–15.7)	32.3	(18.1–47.7)	17.5	(12.5–22.2)
65–74	8.7	(6.8–10.7)	8.6	(6.4–11.1)	24.3	(13.0–38.1)	12.5	(8.8–16.1)
75–100	4.8	(3.7–5.9)	4.8	(3.5–6.2)	14.5	(7.3–24.6)	7	(4.9–9.3)

Note: All PAF values are percentages indicating the proportion of each illness attributable to exposure to racial discrimination and were calculated based on the prevalence data discussed in the text. Values in parenthesis are confidence intervals

**Table 4:** Population attributable fractions: the prevalence of illness attributable to EOD (non-dichotomous) by age and gender

Age group	Illness/Health outcome							
	Depression		Anxiety		PTSD		Psychological disorders	
	PAF (%)	95% CI	PAF (%)	95% CI	PAF (%)	95% CI	PAF (%)	95% CI
Males								
17–24	27.8	(15.4–40.7)	24.8	(19.0–30.8)	29.5	(20.2–39.3)	23.4	(13.2–36.4)
25–34	26.5	(14.6–39.2)	23.6	(18.0–29.4)	28.1	(19.2–37.7)	22.3	(12.5–34.9)
35–44	23	(12.4–34.8)	20.4	(15.4–25.7)	24.5	(16.5–33.4)	19.2	(10.6–30.7)
45–54	21.5	(11.5–32.9)	19.1	(14.4–24.1)	23	(15.4–31.6)	17.9	(9.8–29.0)
55–64	18.1	(9.5–28.3)	15.9	(11.9–20.4)	19.4	(12.7–27.1)	15	(8.1–24.7)
65–74	12	(6.1–19.6)	10.4	(7.7–13.6)	12.9	(8.2–18.6)	9.8	(5.1–16.8)
75–100	9.4	(4.7–15.7)	8.2	(6.0–10.8)	10.2	(6.4–14.9)	7.7	(4.0–13.4)
Females								
17–24	24.5	(13.4–36.7)	21.8	(16.6–27.3)	26.1	(17.7–35.4)	20.5	(11.4–32.6)
25–34	23.5	(12.7–35.4)	20.8	(15.8–26.2)	25	(16.8–34.0)	19.6	(10.8–31.3)
35–44	22.5	(12.1–34.2)	19.9	(15.1–25.1)	24	(16.1–32.8)	18.8	(10.3–30.2)
45–54	21	(11.2–32.3)	18.6	(14.0–23.6)	22.4	(15.0–31.0)	17.5	(9.6–28.4)
55–64	17.8	(9.3–27.9)	15.6	(11.7–20.0)	19	(12.5–26.7)	14.7	(7.9–24.3)
65–74	12.7	(6.5–20.7)	11.1	(8.2–14.4)	13.7	(8.8–19.7)	10.4	(5.5–17.8)
75–100	7.2	(3.5–12.1)	6.2	(4.5–8.2)	7.7	(4.9–11.5)	5.8	(3.0–10.3)

Note: All PAF values are percentages indicating the proportion of each illness attributable to exposure to racial discrimination and were calculated based on the prevalence data discussed in the text. Values in parenthesis are confidence intervals

Tables 4 is the non-dichotomous (3-point and above) exposure measure version of Tables 3, reporting the proportion of the prevalence of the designated illness attributable to the prevalence of EOD among men and women respectively. The values are calculated assuming EOD can be reduced from high to the lowest theoretical minimum. For example, reducing EOD to the lowest possible level can result in a 23.6% reduction in the prevalence of anxiety among men aged 25–34. A similar reduction of EOD can result in a 22.4% reduction in the prevalence of PTSD among women aged 45–54. Likewise, a reduction of EOD to its theoretical minimum, would reduce the prevalence of depression among men aged 25–34 years by 26.5%. This would mean a 26.5% saving in the treatment cost of depression in this age group. The rest of the values can be interpreted similarly.

### Disability Adjusted Life Years (DALYs)

Table 5 reports the final results of our BoD analysis. A total of 235,452 DALYs per year is lost in Australia which can be attributed to the prevalence of EOD. The number of DALYs lost is relatively higher among women (DALY = 140,073) compared to men (DALY = 95,397). Depression for women (32.0%) and psychological disorders for men (33.8%) are the leading causes. For men, depression accounts for 30.3% of lost DALYs attributable to EOD. Overall, the loss in DALYs caused by EOD is estimated to be 8.9% of DALYs from all causes in Australia. This would place EOD above tobacco as a major risk factor since tobacco was estimated to account for 204,788 in lost DALYs (according to the 2003 BoD in Australia, see [47]). A detailed version of this table which disaggregates the findings by age groups is reported in Tables 8 and 9 in Appendix.

**Table 5:** EOD attributable burden of disease (DALYs) by gender and causes in Australia

Cause/Illness	Male (%)	Female (%)	Total
Anxiety	15,890 (16.7)	24,908 (17.8)	40,797
Depression	28,888 (30.3)	44,786 (32.0)	73,673
PTSD	18,366 (19.3)	32,776 (23.4)	51,142
Psychological disorders	32,235 (33.8)	37,604 (26.8)	69,840
Total of illnesses attributable to EOD	95,379 (100)	140,073 (100)	235,452
DALYs from all causes in Australia^a^	1,364,614	1,268,156	2,632,770
Percentage of all DALYs attributable to EOD	7.0%	11.0%	8.9%

Note: The values are DALYs attributable to the prevalence of EOD in a year. The percentages in parentheses indicate DALYs caused by EOD as a proportion of the total

^a^This data was taken from *The burden of disease and injury in Australia 2003* [49]

#### The health costs of racial discrimination

The monetary estimate of the total DALYs attributable to EOD allows for comparison with aggregate economic measures. Table 6 reports the health cost of EOD in Australia, calculated using the value of statistical life (VSL) approach. According to this estimation, EOD accounts for a total of $37.9 billion in health cost to the Australian economy. This is roughly 3.02% of the annual average GDP for the period 2001–11 (for GDP figures see [58]). The largest cost of EOD, $11.9 billion, comes through its effect on depression. This result is comparable with previous studies which used a human capital approach to estimate the cost of EOD. Particularly, the Joint Economic Committee has reported that EOD costed the U.S. economy 4% of GDP while Brimmer [24] estimated the EOD cost to be $241 billion (3.8% GDP).

**Table 6:** Health cost of racial discrimination in Australia ($ millions)

Cause/Illness	Men	Women	Total cost
Anxiety	2557	4009	6566
Depression	4649	7208	11,857
PTSD	2956	5275	8231
Psychological disorders^a^	5188	6052	11,240
Total of illnesses attributable to EOD	15,350	22,543	37,893
Australian average annual GDP (2001–2011)			1,256,769
Health cost of EOD as a percentage of GDP			3.02%

Source (for GDP data): World Bank national accounts data, and OECD National Accounts data files. GDP is in constant local currency unit (LCU)

^a^Excluding anxiety, depression and PTSD

## Sensitivity analysis

To check the robustness of the costs reported in Table 6, we have conducted a sensitivity analysis. We look at three scenarios where we use three VSL values: $3.7 million, $6.65 million and $9.6 million. For each of these VSL values, we varied the underlying discount rates in the 0–10% range. The result, reported in Table 7, indicates a range of health costs due to EOD. At the lower VSL scenario (Panel A) the overall cost of EOD ranges between 1.6 and 1.7% of the average GDP for Australia for the 2001–11 periods. Using the highest discount rate of 10% yields an EOD cost of $19.8 billion while a 0% discount rate results in $21.8 billion. At a 3.3% discount rate the estimated cost is $21.1 billion or 1.7% of GDP.

**Table 7:** Sensitivity analysis: health cost of racial discrimination in Australia ($ millions)

Cause of Illness	DALYs	Discount rate											
		0%	1%	2%	3%	**3.3%**	4%	5%	6%	7%	8%	9%	10%
Scenario A: VSL = $3.7 million													
Anxiety	40,797	3774	3736	3700	3664	**3653**	3629	3594	3560	3527	3494	3462	3431
Depression	73,673	6815	6747	6681	6616	**6597**	6553	6490	6429	6369	6310	6252	6195
PTSD	51,142	4731	4684	4638	4593	**4579**	4549	4505	4463	4421	4380	4340	4301
Psychological disorders^a^	69,840	6460	6396	6333	6272	**6254**	6212	6153	6094	6038	5982	5927	5873
Total of illnesses attributable to EOD	235,452	21,779	21,564	21,352	21,145	**21,084**	20,942	20,742	20,547	20,354	20,166	19,981	19,799
Australian average annual GDP (2005–2012)		1,256,769	1,256,769	1,256,769	1,256,769	**1,256,769**	1,256,769	1,256,769	1,256,769	1,256,769	1,256,769	1,256,769	1,256,769
Health cost of EOD as a percentage of GDP		1.7%	1.7%	1.7%	1.7%	**1.7%**	1.7%	1.7%	1.6%	1.6%	1.6%	1.6%	1.6%
Scenario B: VSL = $6.65 million													
Anxiety	40,797	6783	6715	6650	6585	**6566**	6522	6460	6399	6339	6280	6222	6166
Depression	73,673	12,248	12,127	12,008	11,891	**11,857**	11,777	11,665	11,555	11,447	11,341	11,237	11,135
PTSD	51,142	8502	8418	8336	8255	**8231**	8175	8097	8021	7946	7873	7800	7729
Psychological disorders^a^	69,840	11,611	11,496	11,383	11,273	**11,240**	11,164	11,058	10,954	10,851	10,751	10,652	10,555
Total of illnesses attributable to EOD	235,452	39,144	38,756	38,376	38,004	**37,893**	37,638	37,280	36,928	36,583	36,244	35,912	32,960
Australian average annual GDP (2005–2012)		1,256,769	1,256,769	1,256,769	1,256,769	**1,256,769**	1,256,769	1,256,769	1,256,769	1,256,769	1,256,769	1,256,769	1,256,769
Health cost of EOD as a percentage of GDP		3.1%	3.1%	3.1%	3.0%	**3.0%**	3.0%	3.0%	2.9%	2.9%	2.9%	2.9%	2.9%
Scenario B: VSL = $9.6 million													
Anxiety	40,797	9791	9694	9599	9506	**9479**	9415	9325	9237	9151	9066	8983	8901
Depression	73,673	17,682	17,507	17,335	17,167	**17,117**	17,002	16,840	16,681	16,525	16,372	16,222	16,074
PTSD	51,142	12,274	12,152	12,033	11,917	**11,882**	11,802	11,690	11,579	11,471	11,365	11,261	11,158
Psychological disorders^a^	69,840	16,762	16,596	16,433	16,273	**16,226**	16,117	15,963	15,813	15,665	15,520	15,378	15,238
Total of illnesses attributable to EOD	235,452	56,508	55,949	55,400	54,863	**54,703**	54,335	53,818	53,310	52,812	52,323	51,843	51,371
Australian average annual GDP (2005–2012)		1,256,769	1,256,769	1,256,769	1,256,769	**1,256,769**	1,256,769	1,256,769	1,256,769	1,256,769	1,256,769	1,256,769	1,256,769
Health cost of EOD as a percentage of GDP		4.5%	4.5%	4.4%	4.4%	**4.4%**	4.3%	4.3%	4.2%	4.2%	4.2%	4.1%	4.1%

Note: This table is a sensitivity analysis of the results reported in Table 6 at a range of discount rates for three scenarios: Panel A assuming a value of statistical life of $3.7 million, Panel B $6.65 million, and Panel C $9.6 million. The benchmark discount rate and the associated values are indicated in bold numbers. Values reported in columns 1–11 are in millions of dollars unless indicated otherwise

^a^Excluding anxiety, depression and PTSD

At the medium VSL value of $6.65 million (Panel B), the health cost of EOD ranges between 2.9 and 3.1% of average annual GDP (2001–11) [59]. This is $33 billion at a discount rate of 10% and $39.1 billion at 0% discount rate, for the discount rate of 3.3% the cost is $37.9 billion or 3% of GDP. Similarly, at a high VSL of $9.6 billion (Panel C), the cost is 4.1–4.5% of GDP or $51.4 billion for 10% discount rate and $56.5 billion for 0% discount rate. At a 3.3% discount rate, EOD costs 4.4% of GDP, or $54.7 billion per annum.

It can be concluded that the cost estimation varies depending on the underlying parameters, discount rate and VSL estimates. Using the same discount rate (3.3%), the cost estimate variation across VSL estimates is in the range of $21.1–$54.7 billion (1.7–4.4% of GDP).

## Discussion

In this study, we estimated the economic value of reducing EOD directly from an estimated BoD data. Using a cost of illness method, we measured the PAFs for four key health outcomes (depression, anxiety, PTSD and psychological disorders). Our findings indicate substantial loss in DALYs due to EOD. On the average, Australia loses up to 3.02% of GDP per annum as a result of individuals being exposed to some form of racial discrimination. Gender differences are evident in the DALYs estimated due to the prevalence of both racial discrimination and the illnesses. Empirical evidence shows that the prevalence of psychological illnesses tends to be higher among women than men [58, 60]. In addition, the prevalence of EOD also tends to be higher among women [59, 61, 62], as is evidenced in our data. This corresponded with higher prevalence of mental illnesses, leading to higher values of estimated DALYs.

The cost of illnesses estimates reported in this study reflect cost savings measured against their counterfactuals. They can potentially be realised via measures that can reduce the prevalence of racial discrimination, as a risk factor, to zero or the possible minimum. However, the findings we report should be considered exploratory and indicative. They do not necessarily represent immediately realisable estimates. They are opportunity costs, costs that could be saved by avoiding the need for treating the preventable disease [63]. A range of assumptions are involved in this estimation, the causality of the relationship between EOD exposure and health being the most important.

First, establishing causal relationship between EOD and health outcomes from cross-sectional studies has limitations as a target could be experiencing negative health outcomes due to multiple factors. Reverse causation between EOD and negative health outcomes cannot be ruled out although there is considerable evidence suggesting that EOD precedes ill-health [14, 64]. Obtaining association data in terms of RR rather than OR should improve the accuracy of the findings and solve the causality issue. Second, only unadjusted associations between EOD and health are utilised in this analysis. However, confounding due to multivariate effects in such associations cannot be ruled out. For example, factors such as demographic and socio-economic factors can also have a role in EOD and health outcomes. A thorough investigation of longitudinal analysis and adjusted associations should, therefore, give a better picture regarding the BoD outcomes related to the EOD.

Our estimation of DALYs also has some limitations. First, our study has estimated the cost of EOD for only four illnesses although the literature indicates association with physical illnesses such as hypertension, diabetes and hypercholesterolemia [65]. This is likely to lead to the underestimation of the health cost estimates reported. The main reason for the exclusion of physical illnesses in this study is insufficient data for the calculation of BoD estimates. Apart from the four illnesses indicated in Table 5, we could not use 21 associations we analysed in a meta-analysis for they are not available in the Global Burden of Disease (GBD). Either they are only risk factors which cannot be strictly considered illnesses (e.g., overweight, obesity etc.) or they are not defined in the GBD due to co-morbidity (e.g., stress, psychological distress, internalizing symptoms etc.). For those which are not in the GBD categories no disability weight data is available for the calculation of DALYs. For mental/psychological disorder, a composite disability weight index was used by averaging across all the mental disorders weights available in the GBD, Vos & Mathers [66] and Begg et al. [47].

Second, we could not find prevalence data for some illnesses (e.g., stress, internalizing symptoms etc.). Therefore, the requirement for the calculation of DALYs could be completed for just four studies. The association data was obtained from our recent meta-analysis of studies [14] conducted at various times with prevalence data for the period 2001–11. Therefore, there may be confounding across different time periods. The DALYs are crudely measured without accounting for time lag, and adjustments for any confounders. Further refinement and the inclusion of relevant associations are needed to give definitive conclusion regarding the DALYs caused by EOD.

Furthermore, the data used in the calculation are heterogeneous. Prevalence data for anxiety and psychological disorder was obtained from the National Survey of Mental Health and Wellbeing [67]. For depression and PTSD, the prevalence rate from the same source is aggregated by gender. This was disaggregated across age groups using census data, based on the information that depression has similar variation across age groups up to 64 and declining thereafter for those aged 65 and over [68]. In addition, disability weights were obtained from a range of studies including the GBD [69], Vos & Mathers [66], the 2005 Victorian Burden of Disease Study, and Begg et al. [47]. The derivation of these weights, particularly for psychological disorders, involved averaging across studies and disease categories. For example, the GBD [69] disaggregates the weights by age group; Vos & Mathers [66] provide weight range while a single weight is used in the Victorian BoD.

Our estimation does not include direct healthcare expenditures and indirect costs associated with racial discrimination which can arise from inefficiency in the labour market due to the underutilisation of education, skills and experiences of the targets. As such, even for the few health outcomes for which there was the requisite data, our estimates are lower bound as they only measure intangible costs.

Therefore, the total cost is likely to be higher than 3.02% if all cost components were included. A fuller costing of EOD would include hospitalisation and out-of-pocket expenses in addition to the BoD-based intangible cost reported in Table 6. According to the AIHW, Australians (individuals), on the average covered 17.3% of the total health expenditure in 2000–2012 [70]. The rest of the health cost was funded by state and federal governments and private insurers. The AIHW [71] reports that out of pocket expenses accounted for 18.2% of total health spending, and averaged at 2.4 to 2.8% of total household spending in the decade ending 2009. ‘More than half of non-government funding (58%) came from out-of-pocket payments by individuals. This included circumstances where individuals met the full cost of goods or services, as well as where they shared the cost, for example, with private health insurance funds or the Australian Government through Medicare’ (p.475). According to this report, the total out-of-pocket spending by individuals covered $7.7 billion or 47% of the total cost of medications in the years 2009–10. Addition of such costs attributable to EOD exposure can therefore substantially increase the health cost of EOD.^2^

Finally, this study focused on Australian data. However, the analysis can be replicated cross-nationally with potentially comparable findings. The exact cost of EOD for each country will depend on the prevalence rate of EOD, with countries that exhibit higher EOD prevalence also bearing higher overall cost.

## Conclusion

We measured the economic impact of EOD in Australia from the societal perspective. Economic costs are usually estimated based on production loss and/or the consumption of resources. Another important cost component is the pain and suffering involved due to illness that can be attributed to a risk factor, such as racial discrimination. In this study, we found the BoD attributable to EOD associated with anxiety, depression, PTSD and psychological disorders to be substantial. Overall, the four illnesses accounted for 235,452 DALYs lost in Australia due to EOD of which depression accounted for 31.3% of the total DALYs.

In monetary terms, for a VSL of $6.65 million, a time period of 40 years and a discount rate of 3.3%, which is roughly $166,250 per DALY, we estimated the cost of EOD related to the four health outcomes to be $37.9 billion, which is roughly 3.02% of the average annual GDP (2001–2011) of Australia [72]. This is proportional to previous findings in U.S. studies that are based on the human capital approach.

Using new evidence and integrating it with advances in public health research, our study was able to estimate the economy-wide loss directly attributable to EOD. The evidence clearly shows that the Australian economy would be significantly better off in the absence of EOD. In addition to its infringement on the rights of individuals and groups, racial discrimination has detrimental impact on the economy. The cost would even be much higher if discrimination related direct expenditures were added. As such, the addition of hospitalisation and out-of-pocket expenditure should also be estimated in future work to give a better picture of the total health cost of EOD. The other source of cost is the indirect costs related to loss of productivity. As there exists little research that has estimated this, more research is needed in this area to corroborate the scant evidence base regarding the cost of EOD.

In summary, although we did not include a range of cost items in our analysis, our study was able to estimate a vital aspect of the cost of racial discrimination. We found that racial discrimination is substantially costly to the health of individuals expressed in loss of healthy life years. The findings of this study are particularly important in informing public policies and advocacy activities related to public health, community social cohesion, anti-discrimination and cultural diversity. By quantifying the cost of discrimination, the study contributes to the rationale for anti-racism strategies that seek to benefit society by reducing the costs associated with discrimination. The findings also indicate that given some of the costs resulting from EOD are avoidable, measures taken by governmental and non-governmental institutions to curb racial discrimination are likely to be socially and economically feasible. Countries with racially and ethnically diverse population can therefore realise substantial savings by enforcing effective anti-discrimination measures [73].

## Appendix

**Table 8:** EOD attributable burden of disease (DALYs) for men in Australia by age and causes

Type of Illness/Health outcome	Age category	Prevalent cases	Disability weight	PAF	DALYs
Anxiety	17–24	92,660	0.149	0.200	2759
	25–34	125,395	0.149	0.190	3542
	35–44	176,308	0.149	0.162	4264
	45–54	150,582	0.149	0.151	3398
	55–64	79,335	0.149	0.126	1484
	65–74	30,116	0.149	0.081	364
	75–100	8473	0.149	0.063	80
Depression	17–24	90,907	0.283	0.200	5145
	25–34	125,465	0.283	0.190	6735
	35–44	131,571	0.283	0.162	6048
	45–54	123,803	0.283	0.152	5310
	55–64	99,971	0.283	0.126	3554
	65–74	61,090	0.283	0.081	1404
	75–100	38,529	0.283	0.063	692
PTSD	17–24	69,178	0.096	0.459	3045
	25–34	95,475	0.096	0.442	4054
	35–44	100,122	0.096	0.396	3811
	45–54	94,210	0.096	0.377	3410
	55–64	76,075	0.096	0.327	2391
	65–74	46,488	0.096	0.23	1028
	75–100	35,090	0.096	0.187	629
Psychological disorders	17–24	296,300	0.224	0.125	8289
	25–34	321,500	0.224	0.118	8490
	35–44	319,000	0.224	0.100	7122
	45–54	262,100	0.224	0.093	5433
	55–64	126,500	0.224	0.076	2148
	65–74	53,800	0.224	0.048	579
	75–100	20,900	0.224	0.037	174
Total					95,379

Note: DALYs are estimated using the standard formula (3) for each illness by age group

**Table 9:** EOD attributable burden of disease (DALYs) for women in Australia by age and causes

Type of Illness/Health outcome	Age category	Prevalent cases	Disability weight	PAF	DALYs
Anxiety	17–24	206,467	0.149	0.174	5359
	25–34	226,359	0.149	0.166	5600
	35–44	248,614	0.149	0.159	5878
	45–54	235,276	0.149	0.148	5171
	55–64	122,097	0.149	0.123	2241
	65–74	39,403	0.149	0.086	508
	75–100	21,035	0.149	0.048	150
Depression	17–24	143,311	0.283	0.174	7071
	25–34	205,822	0.283	0.166	9680
	35–44	220,309	0.283	0.159	9902
	45–54	207,824	0.283	0.148	8683
	55–64	165,160	0.283	0.123	5761
	65–74	105,170	0.283	0.087	2575
	75–100	82,283	0.283	0.048	1113
PTSD	17–24	122,265	0.096	0.417	4895
	25–34	175,596	0.096	0.403	6794
	35–44	187,956	0.096	0.39	7038
	45–54	177,304	0.096	0.37	6294
	55–64	140,905	0.096	0.323	4364
	65–74	89,726	0.096	0.243	2092
	75–100	93,117	0.096	0.145	1299
Psychological disorders	17–24	374,800	0.224	0.108	9032
	25–34	376,800	0.224	0.102	8622
	35–44	397,500	0.224	0.097	8663
	45–54	351,600	0.224	0.09	7087
	55–64	190,200	0.224	0.074	3165
	65–74	70,000	0.224	0.051	804
	75–100	36,800	0.224	0.028	230
Total					140,073

Note: DALYs are estimated using the standard formula (3) for each illness by age group

## Abbreviations

BoD: Burden of disease
CRP: Challenging Racism Project
DALY: Disability adjusted life years
DFO: Dual-frame Omnibus Survey
EOD: Experience of racial discrimination
GBD: Global burden of disease
GDP: Gross domestic product
MSC: Mapping Social Cohesion
OR: Odds ratio
PAF: Population attributable fraction
PTSD: Post-traumatic stress disorder
RR: Risk ratio
VSL: Value of statistical life
WHO: World Health Organisation

## Footnotes

1 The inclusion criteria which can be found in Paradies et al. [14] include:

1. be published in a journal article or dissertation
2. report at least one association between racism and a health outcome/s
3. be an empirical study
4. report quantitative data
5. contain relevant exposure/s and/or outcome/s
6. report unadjusted associations
7. report statistics that allow calculation of effect size

For this paper, further inclusion criteria were added:

1. The study has to report at least one of four health outcomes (depression, anxiety, PTSD and psychological disorder).
2. It has to report statistics/data that allow for the calculation of OR.

2 Another source of cost would be litigation cost that results if alleged racial discrimination is brought to the court. Some of the settlement and litigation cost would be transfer payment and may not be attributed as loss in GDP. But, some part would be incurred as a loss to society as time and resources are diverted to execute the litigation process. An example for this is the $72.69 million per annum lost in litigation cost to settle racial discrimination complaints in the U.S. (see: a 2014 Equal Employment Opportunity Commission report).
